# Alternate fluency in Parkinson’s disease: A machine learning analysis

**DOI:** 10.1371/journal.pone.0265803

**Published:** 2022-03-23

**Authors:** Roberta Ferrucci, Francesca Mameli, Fabiana Ruggiero, Mariella Reitano, Mario Miccoli, Angelo Gemignani, Ciro Conversano, Michelangelo Dini, Stefano Zago, Silvie Piacentini, Barbara Poletti, Alberto Priori, Graziella Orrù

**Affiliations:** 1 Department of Health Sciences, Aldo Ravelli Research Center, University of Milan, Milan, Italy; 2 ASST-Santi Paolo e Carlo Hospital, Milan, Italy; 3 IRCCS Ca’ Granda Foundation, Policlinico of Milan, Milan, Italy; 4 Department of Clinical and Experimental Medicine, University of Pisa, Pisa, Italy; 5 Department of Surgical, Medical, Molecular & Critical Area Pathology, University of Pisa, Pisa, Italy; 6 IRCCS Foundation -Neurological Institute Carlo Besta, Milan, Italy; 7 Auxologico Italian Institute–IRCCS, Milan, Italy; University of Toronto, CANADA

## Abstract

**Objective:**

The aim of the present study was to investigate whether patients with Parkinson’s Disease (PD) had changes in their level of performance in extra-dimensional shifting by implementing a novel analysis method, utilizing the new alternate phonemic/semantic fluency test.

**Method:**

We used machine learning (ML) in order to develop high accuracy classification between PD patients with high and low scores in the alternate fluency test.

**Results:**

The models developed resulted to be accurate in such classification in a range between 80% and 90%. The predictor which demonstrated maximum efficiency in classifying the participants as low or high performers was the semantic fluency test. The optimal cut-off of a decision rule based on this test yielded an accuracy of 86.96%. Following the removal of the semantic fluency test from the system, the parameter which best contributed to the classification was the phonemic fluency test. The best cut-offs were identified and the decision rule yielded an overall accuracy of 80.43%. Lastly, in order to evaluate the classification accuracy based on the shifting index, the best cut-offs based on an optimal single rule yielded an overall accuracy of 83.69%.

**Conclusion:**

We found that ML analysis of semantic and phonemic verbal fluency may be used to identify simple rules with high accuracy and good out of sample generalization, allowing the detection of executive deficits in patients with PD.

## Introduction

Neuropsychological tests are used for clinical assessment of patients with Parkinson’s disease (PD). Neuropsychological testing has become more accurate in assessing peculiar segments of cognitive impairment in specific neurological populations (i.e., investigating patterns of anterograde memory to predict different aetiologies of dementia) [1], rather than evaluating generic aspects of cognitive behavioural functioning [2]. Accurate and highly related cognitive profiles are required for specific disorders in order to detect precise cognitive dysfunctions and plan individualised treatments in a variety of neurological populations. This would be particularly useful in individuals with PD because although changes of cognitive functions in PD patients have been extensively documented and defined as a frontal-type executive dysfunction [3], the analysis of specific executive components and subcomponents results has been inconsistent. This has been described by Kudlicka et al. [4] in their systematic review and meta-analysis. Furthermore, the pattern of executive impairment in PD is still debated [5, 6].

In general terms, the efficiency of executive functions is age-related [7, 8] and the components of the executive domain might be differentially affected by brain lesions in neurological populations [9]. Extra-dimensional set-shifting refers to the ability to regulate one’s attention and behaviour, by switching between different stimuli dimensions and response sets, based on environmental contingencies or explicit instructions [10, 11]. An increasing number of studies have reported that dysfunction in attention commonly appears in connection with complex tasks that require spatial and non-spatial shifting [12], in addition to sustained attention [13]. The underlying pathological mechanisms of this dysfunction remain unclear; however, studies have indicated the involvement of abnormal metabolic activity and cholinergic deficits in the thalamus [14, 15]. An fMRI study of non-spatial shifting of selective attention has reported that the cerebellar and parietal regions may be of importance [12].

Recently, a new test to assess set-shifting abilities has been introduced by Costa et al. [1]. This test combines phonemic and semantic fluencies and is an alternate phonemic/semantic task with standardized and normative data from the Italian population. Additionally, as the authors highlighted in their study, from the alternate fluency test is possible to derive an index, named shifting index, which represents an indicator of set-shifting abilities, which require frontal cortex cognitive processing. This test could be a useful and reliable tool for individuals with fronto-striatal related disorders, such as PD, because there are no high demands on motor systems. In contrast, other tests may be unreliable in this population of individuals because they depend on reaction times or motor performance.

The aim of the present study was to investigate whether individuals with PD showed changes in their level of performance in extra-dimensional shifting by implementing a novel analysis method. For this reason, we applied Machine Learning (ML) analysis to examine verbal fluency and set-shifting abilities measured by the semantic, phonemic, and the new alternate phonemic/semantic fluency test in a sample of patients with PD.

Psychometric techniques have been recently benefitted from the use of ML in order to fine tune the predictive capacity of the test [16–19]. This approach, applied to psychometrics, is aimed to maximise generalizability, focusing primarily on cross-validated results and prediction rather than on fitting statistical models to data, which is, by contrast the main focus of Item Response Theory and Classical Test Theory [20, 21] and could represent an alternative and effective way of detecting accurate rules that enable the detection of set-shifting deficits in patients with PD.

The classical fitting-first procedure frequently results in overfitting, with the consequence that non-cross-validated result can be rarely replicated. Recent studies have shown that psychometric testing may be boosted by using machine learning (ML) techniques [22]. These techniques have been used to develop classification models in forensic psychology [17, 19], neuroimaging [23], genetics [24], and clinical medicine [25].

## Materials and methods

### Participants

We recruited 140 Italian-speaking participants (95 men and 45 women; mean age = 63.50 ± 10.64 years, mean education = 12.16 ± 3.89 years). Participants were patients with idiopathic PD (disease duration range = 3–20 months) who had been diagnosed according to UK Brain Bank criteria by a team of expert neurologists through anamnestic interviews, neurological examinations, neuropsychological testing, and neuroimaging tests. Subjects were ON medication during the neuropsychological assessment. An inclusion criterion for the study was the absence of dementia, as measure.

### Assessment

The participants were evaluated by experienced neuropsychologists working in the neuropsychology outpatient clinics of the participating hospitals, and the whole assessment lasted about one hour.

The neuropsychological assessment included the following tests: Mini Mental State Examination (MMSE) [21] or Montreal Cognitive Assessment (MOCA) [22], Phonemic (FAS) [23, 24], Semantic [25], and Alternate Fluency test [14].

In order to assess the *Global cognitive functioning*, participants underwent to the MMSE and MoCA. The MMSE or Folstein test [21] and MoCA [22] are 30-point questionnaires that are used extensively in clinical and research settings to measure global cognitive impairment.

Verbal fluency was assessed though the administration different three *Verbal Fluency Tests*. Verbal fluency tasks assess verbal ability and executive control of verbal fluency performance. In the Phonemic Fluency (PF) test, participants are instructed to produce as many words as possible beginning with the letters: ‘A,’ ‘F,’ or ‘S’ [24] (one minute for each trial).

In the Semantic Fluency (SF) test, participants are instructed to list as many words as possible belonging to specific categories: ‘colours,’ ‘animals,’ or ‘fruits’ [25] (one minute for each trial).

In the Alternate Phonemic/Fluency (AF) test, participants are required to alternate letter-cued words with category-cued words as follows (one minute for each trial): letter ‘A’ and ‘Colours;’ letter ‘F’ and ‘Animals;’ or letter ‘S’ and ‘Fruits’ [14]. Letter-cued words and category-cued words do not have to be related to each other in the AF test; the only requirement is that participants alternate a letter-cued word (of any kind, following the rules of the PF test) and a category-cued word (regardless of the initial). Through the AF test it is possible to derive a specific measure called “shifting index”. The shifting index is computed by taking into account the words generated in all three subtests (PF, SF, AF) according to the following formula: total words generated in the AF subtest/[(PF score + SF score)/2] (as indicated by Costa et al. [14], p. 5).

### Experimental procedures

The first test administered was the MMSE or MOCA. The three fluency tests were administered consecutively in the following order: phonemic, semantic, and then alternate. Participants were instructed not to use proper nouns, conjugate verbs, or repeat the same word with a different ending in the fluency tests. The number of words produced in one minute was recorded for each trial of the verbal fluency tests. Patients were required to carry out all the tests with their maximum effort.

All subjects provided informed consent. The protocol and procedures were approved by the institutional review board. The procedures were conducted in accordance with the Declaration of Helsinki.

### Data analysis

All statistical analyses were performed using R 3.5.0 and Weka 3.8 [26].

The Kolmogorov-Smirnov test was used to verify the normal distributions of the data. Variables that displayed Gaussian distribution were analysed using linear regression and calculation of Pearson’s correlation coefficient. The ranges of the coefficient were considered as: 0.0 to −0.29 (inverse low correlation); −0.3 to −0.69 (inverse moderate correlation); and −0.7 to −1 (inverse high correlation). Only coefficients associated with significant p-values were reported in the results. The significance level of α was set to 0.05.

We used k-fold cross validation to evaluate the classification accuracy on the AF test performances. This method avoids overfitting, i.e., abnormal model fitting that can be prevented using out-of-sample accuracy estimation as a proxy for in-field accuracies. It has been shown that k-fold cross validation can estimate true accuracies in small samples.

## Results

### Statistical analysis

Means, standard deviations, age range, education, and test scores are reported in Table 1.

**Table 1:** Demographic characteristics and test performance.

	Mean	Std. Dev.
Age	63.38	10.740
Education	12.17	3.896
MMSE/MOCA	25.82	3.779
PF	33.472	12.2337
SF	43.0878	10.19730
AF	28.636	11.7616
Shifting Index	0.6911	0.24613

*Notes*: *AF*, Alternate Fluency Test; *PF*, Phonemic Fluency test; *SF*, Semantic Fluency Test; *Shifting*, shifting index.

**Fig 1:**
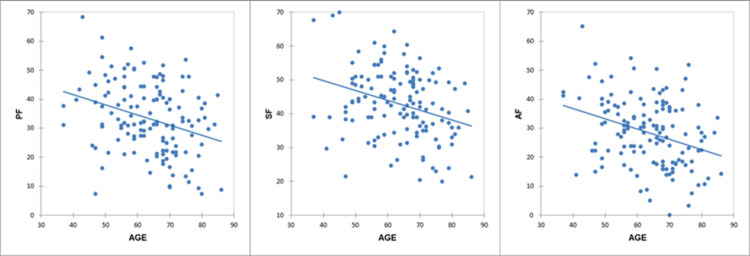
Correlations between age and fluency tests scores. Age and phonemic fluency (r = −0.31, p = 0.0002); age and semantic fluency (r = −0.3, p = 0.0003); age and alternate fluency (r = −0.32, p < 0.0001). *PF*, Phonemic Fluency; *SF*, Semantic Fluency; *AF*, Alternate Fluency.

Age was inversely correlated with PF (r = −0.31, p = 0.0002), SF (r = −0.3, p = 0.0003), and AF (r = −0.32, p < 0.0001) scores, as shown in Fig 1.

Additionally, PF was significantly higher in females when compared with males (36.5 ± 9.9 *vs*. 32 ± 12.9, p = 0.029).

### Correlations between age and alternate fluency stratified for MMSE or MOCA

**Fig 2:**
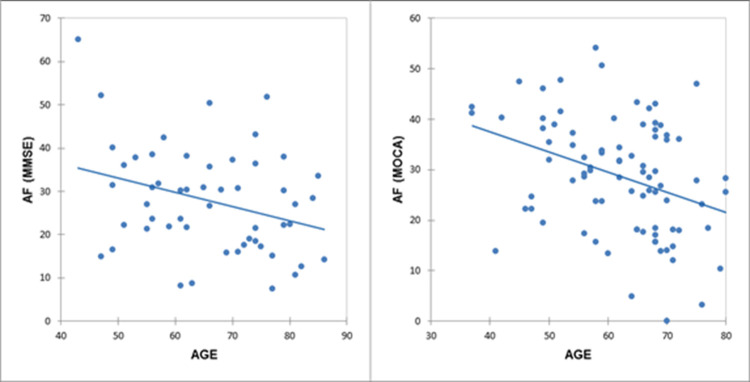
Correlations between age and alternate fluency with the sample stratified for MMSE or MOCA. AF/MMSE: r = −0.28, p = 0.04; AF/MOCA: r = −0.35, p = 0.001. *AF*, Alternate Fluency; *MMSE*, Mini Mental State Examination; *MOCA*, Montreal Cognitive Assessment.

To gain a greater understanding of the performance of patients with PD on the new test, AF, we stratified the sample based on AF and MMSE or MOCA scores. Interestingly, we found significant inverse correlations between age and AF when stratified for MMSE or MOCA (AF/MMSE: r = −0.28, p = 0.04; AF/MOCA: r = −0.35, p = 0.001; Fig 2).

**Fig 3:**
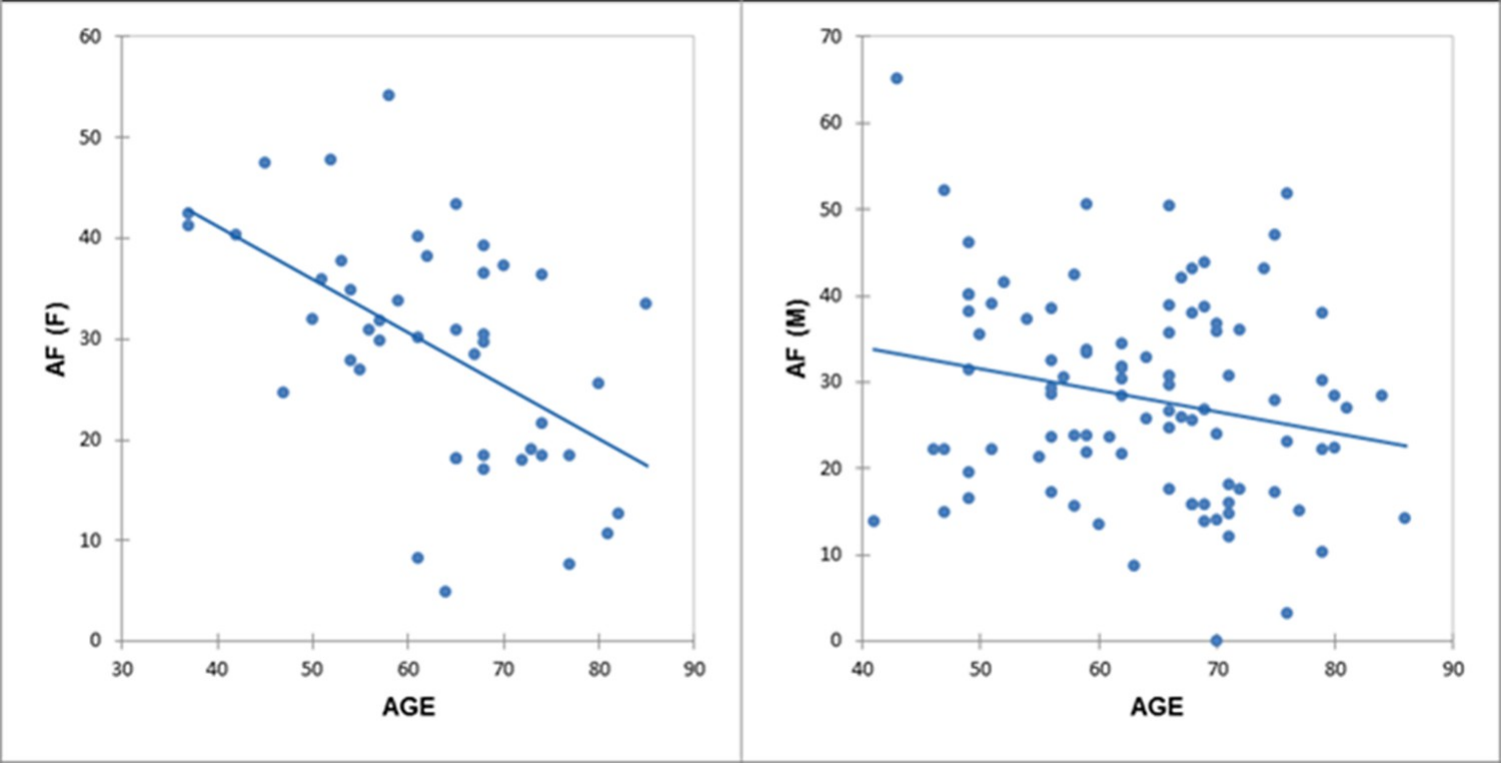
Correlations between age and alternate fluency with the sample stratified for gender. AF/females: r = −0.53, p = 0.0002; AF/males: r = −0.2, p = 0.38. *AF*, Alternate Fluency; *F*, Females; *M*, Males.

**Fig 4:**
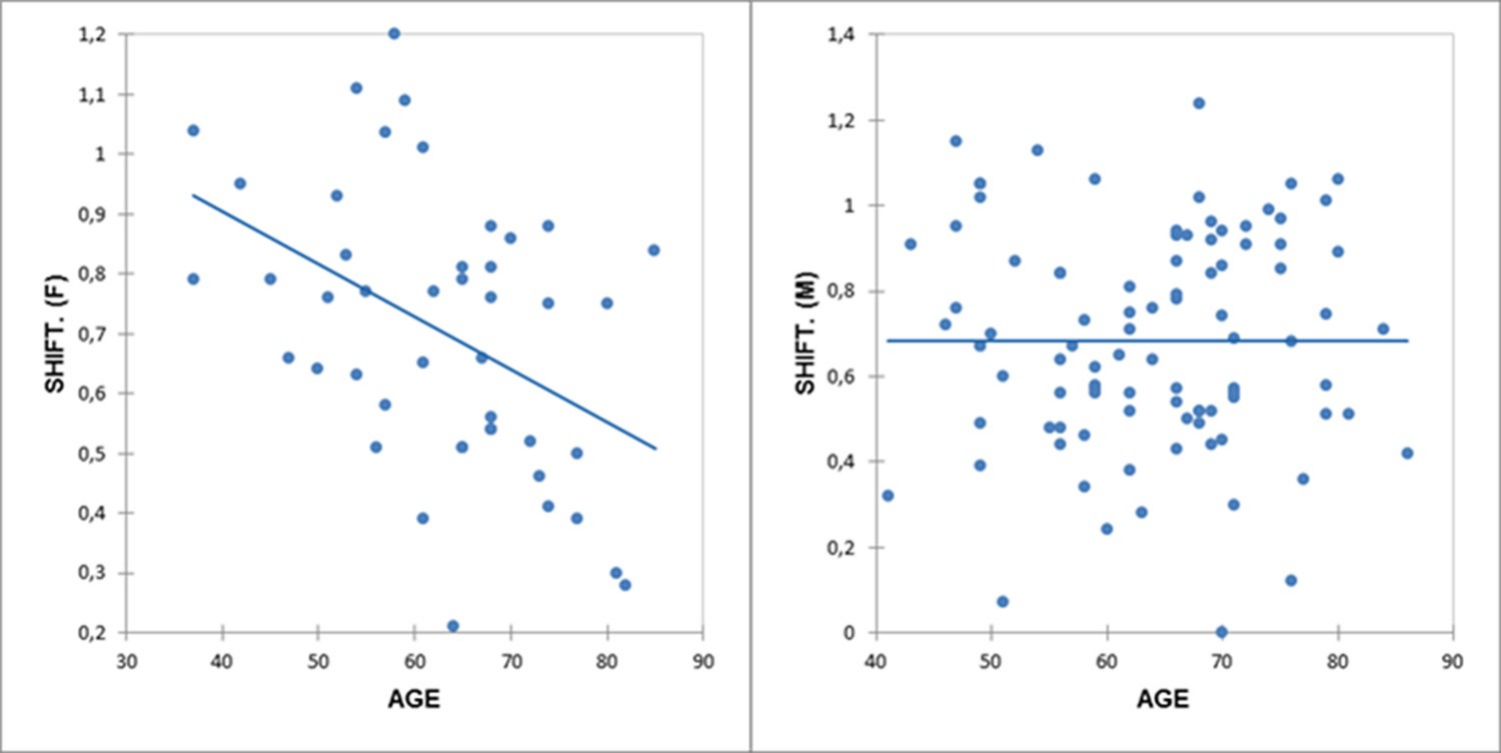
Correlations between age and shifting index in the sample stratified for gender. The shifting index is significant for females alone (r = −0.44, p = 0.003). *F*, Females; *M*, Males; *SHIFT*, shifting index.

Analyses stratified according to male/female sex revealed the following significant results (Fig 3): AF/females (r = −0.53; p = 0.0002) and shifting index/females (r = −0.44; p = 0.003) (Fig 4). Interestingly, the inverse correlations with AF were stronger in females than in males (r = −0.2; p = 0.38).

In contrast with Costa et al. [1], the shifting index in males was not significantly correlated with age. This suggests that male patients with PD do not display a typical reduction in the shifting index that is observed in control participants and female patients with PD. No significant correlations were found between education and test scores in the sample or subgroups.

Taken together, these correlational analyses highlighted that verbal fluencies (phonemic, sematic, and alternate) were cognition- and age-dependent. Therefore, global cognitive status (as measured by MMSE and MOCA) decreases as age increases, which reduces performance on all verbal fluency tests.

### Machine learning based classification accuracies of high and low performers on alternate fluency

To investigate the independent variables that correlate with AF test performance in patients with PD, we divided the participants (N = 140) into two subgroups: low-AF (L-AF) and high-AF (H-AF) performers. Next, we performed ML analysis to evaluate the classification accuracy between L-AF and H-AF [26] performers.

ML classifiers underperform in unbalanced data samples; therefore, we balanced the number of participants in the two AF performance subgroups. Based on a median AF score = 26 for the total sample, we retained the top 46 performers (H-AF; n = 46, AF score range = 32–72) and bottom 46 performers (L-AF; n = 46, AF score range = 0–20). Those who scored closer to the median score (n = 48, AF score range = 21–31) were excluded. Table 2 shows the main features of the two subgroups (L-AF + H-AF).

**Table 2:** Demographic characteristics and performance on the administered tests for the Low-AF and High-AF performers.

	Low-High Performers (n = 92)
	Low-performers (n = 46)
	High-performers (n = 46)
	(M = 61; F = 31)
Age (mean±st.dev.)	63.60±11.32
Education (mean±st.dev.)	12.29±3.84
MMSE/MOCA (mean±st.dev.)	25.33±4.16
PF (mean±st.dev.)	34.81±15.22
SF (mean±st.dev.)	40.60±13.29
AF (mean±st.dev.)	26.76±15.91
Shifting Index (mean±st.dev.)	0.65±0.28

Notes: *AF*, Alternate Fluency Test; *PF*, Phonemic Fluency test; *SF*, Semantic Fluency Test.

### Correlation

Next, we assessed which independent variables (gender, age, education, MMSE/MOCA, phonemic fluency, semantic fluency) had the strongest correlation with AF test scores and the derived shifting index. Table 3 shows the ranked attributes and correlations.

**Table 3:** Correlation analysis between Alternate Fluency test score and other neuropsychological scores and demographic variables.

Ranked attributes	Correlation
Semantic Fluency	0.75*
Phonemic Fluency	0.74*
Age	0.51*
MMSE/MOCA	0.43*
Education	0.22
Gender	0.11

*p-value < 0.05.

### Independent variable analysis based on the correlations as predictors of the two classes

The parameter that demonstrated maximum efficiency (removing the obvious variables scores, such as the AF test and derived shifting index) in classifying the participants as L-AF or H-AF performers was the SF test. The cut-off was identified using oneR [27], a 1-level classification algorithm that uses the minimum-error attribute for prediction by discretizing numeric attributes. This yielded an accuracy of 86.96% (AUC = 0.87; d = 1.59; F1 = 0.87). The optimal rule was as follows:

*if the SF test score is <41.5, then the participant is classified as a low performer on alternate fluency*;

*and*

*if the SF test score is ≥41.5, then the participant is classified as a high-performer on alternate fluency*.

The reported decision rule is not the best classifier; however, it provides a better understanding of the principle, which results in high accuracy in classifying low- and high-performers on the AF test, as shown in Table 4. It is clear from the confusion matrix that L-AF and H-AF are identified with high accuracy.

**Table 4:** Confusion matrix with the best performing single decision rule with oneR using 10-fold cross-validation.

	L-AF (n = 46)	H-AF (n = 46)	Accuracy for corrected classification
L-AF	**39**	7	85%
H-AF	5	**41**	89%

AUC = 0.87; d = 1.59; F1 = 0.87. The rule was based on SF test scores and was the following: *if the semantic fluency test score is <41*.*5 or ≥41*.*5*, *then the participant is classified as a low- or high-performer*, *respectively*. *L-AF*, low-AF performers on the alternate fluency test; *H-AF*, high-AF performers on the alternate fluency test.

### The best independent variables that maximize classification accuracy

A features selection was performed using Weka 3.8 [26] to identify the best independent variables to be entered in ML models. This widely-used process allows the removal of irrelevant features; redundant features in a dataset can decrease the accuracy of the model and facilitate learning based on irrelevant features. The benefits of using this procedure include a reduction in overfitting; accuracy improvement; and an increase in model generalization [28]. Therefore, the dimensionality of training and test data was reduced using attribute selection before being passed on to a classifier.

The features we used to develop the ML models were age, education, gender, MMSE/MOCA, and phonemic and semantic tests. These six features were entered into different ML classifiers that were trained to classify every subject as belonging to L-AF or H-AF. We selected the following classifiers to obtain a representative set of different categories: Naïve Bayes, Logistic Regression, Simple Logistics, Support Vector Machine, and Random Forest [29]. Each classifier was run using 10-fold cross validation. The cross-validation process was repeated with each one of the ten subsamples used as a validation set (ten repetitions). The mean of these results was calculated to produce the classification accuracy estimation. Primarily, this procedure is used when the goal is prediction. This process can estimate the accuracy of a predictive model. The results and accuracy scores between the different classifiers, measured as % correct, AUC, and F1, are reported in Table 5.

**Table 5:** Accuracies as measured by % correct, AUC and F1 obtained by five different ML classifiers.

Classifier	Accuracy (%)	AUC (Cohen’s d)	F1
Naïve Bayes	88.04%	0.97 (d = 2.66)	0.88
Logistic Regression	89.13%	0.95(d = 2.33)	0.89
Simple Logistics	86.96%	0.94 (d = 2.20)	0.87
Support Vector Machine	89.13%	0.89 (d = 1.73)	0.89
Random Forest	85.87%	0.96 (d = 2.48)	0.86

Notes: perfect classification of exemplars in the two categories has an AUC of 1 and F1 of 1. AUC is defined as Area Under the Curve in ROC analysis and F1. To compare AUC with the best-known effect size, Cohen’s d is included.

All the classifiers yielded similar accuracy results (range, 86.9–89.13%).

### Classification accuracy analysis based on the following preselected features: Age, education, MMSE/MOCA, and phonemic fluency

To evaluate the classification accuracy of L-AF and H-AF based on the PF test, we considered the following features as predictors: age, education, MMSE/MOCA, and PF test score alone.

Following removal of the SF test, the PF test cut-offs were identified using oneR, which yielded an overall accuracy of 80.43% (AUC = 0.80; d = 1.19; F1 = 0.80). Cut-offs of <30.5 and ≥30.5 correctly classified 85% of patients in the L-AF and 76% of patients in H-AF subgroups, respectively. Results and accuracy sores between classifiers, measured by % correct, AUC, and F1, are reported in Table 6.

**Table 6:** Accuracies as measured by % correct, AUC and F1 obtained by five different ML classifiers.

Classifier	Accuracy (%)	AUC (Cohen’s d)	F1
Naïve Bayes	84.78%	0.94 (d = 2.19)	0.85
Logistic Regression	86.96%	0.93 (d = 2.08)	0.87
Simple Logistics	88.04%	0.92 (d = 1.98)	0.88
Support Vector Machine	85.87%	0.86 (d = 1.52)	0.86
Random Forest	81.52%	0.92 (d = 1.98)	0.81

Perfect classification of exemplars in the two categories has an AUC of 1 and F1 of 1. AUC is defined as Area Under the Curve in ROC analysis and F1. To compare AUC with the best-known effect size, Cohen’s d is included.

### Classification accuracy based on the shifting index

To evaluate the classification accuracy of L-AF and H-AF based on the shifting index, we considered the following features as predictors: age, education, MMSE/MOCA, and shifting index alone.

The shifting index cut-offs were identified using oneR, which yielded an overall accuracy of 83.69% (AUC = 0.84; d = 1.40; F1 = 0.84). Cut-offs of <0.55 and ≥0.55 correctly classified 76% of patients in the L-AF and 91% of patients in the H-AF subgroups, respectively. Results and accuracy scores between classifiers, measured by % correct, AUC, and F1, are reported in Table 7.

**Table 7:** Accuracies as measured by % correct, AUC, and F1 obtained by five different ML classifiers.

Classifier	Accuracy (%)	AUC (Cohen’s d)	F1
Naïve Bayes	89.13%	0.94 (d = 2.19)	0.89
Logistic Regression	81.52%	0.90 (d = 1.81)	0.81
Simple Logistics	88.04%	0.92 (d = 1.98)	0.88
Support Vector Machine	88.04%	0.88 (d = 1.66)	0.88
Random Forest	86.96%	0.93 (d = 2.08)	0.87

Notes: perfect classification of exemplars in the two categories has an AUC of 1 and F1 of 1. AUC stands for Area Under the Curve in ROC analysis and F1. To compare AUC with the best-known effect size, Cohen’s d is included.

## Discussion and conclusions

Our study found similar results to the original study by Costa et al. (1). For example, we have shown that males exhibited a worse performance on all verbal fluency measures when compared with females. Furthermore, correlational analysis highlighted that verbal fluency performance (phonemic, sematic, and alternate) was dependent on both global cognitive status and age. As predicted, global cognitive status, as measured by MMSE and MOCA, decreases as age increases. This leads to reduced performance on all verbal fluency tests. However, in contrast to Costa et al. [1], we found no significant correlations between shifting index and age in males. Males and females have been shown to activate different prefrontal areas during set-shifting tasks [30], which could explain the different effect of age on shifting index in our study. Furthermore, no significant correlations were found between education and verbal fluency scores. It is possible that education did not correlate with verbal fluency because the educational level of our sample was high and homogeneous (12.29±3.84 years); a correlation could be more easily detected in samples with a higher degree of variability in terms of education.

Our study focused on the AF test as a measure of extradimensional set-shifting. The shifting index is an indicator of set-shifting ability, a high-order element of executive functioning which reflects basal ganglia and frontal cortex cognitive processing, and is thought to be compromised earlier in the course of PD, compared to other executive functions such as planning [10]. Previous studies have reported that efficiency in the executive domain significantly decreases with physiological ageing and neurodegenerative disorders, such as PD, which is confirmed in this study. Evidence also indicates that deficits of extra-dimensional set-shifting might reflect alterations of the norepinephrine system, and not of the dopaminergic system [31]. This confirms the involvement of the locus coeruleus in the neuropathology of PD and suggests the existence of a parallel pathway to cognitive dysfunction. ML analysis of the AF test allowed us to identify that not all individuals with PD exhibit executive difficulties such as set-shifting deficits. It is clinically relevant to assess if patients display dysexecutive vulnerability, since it has been associated with increased risk of global cognitive decline [32] and reduced quality of life [33]. In this study, we found that ML analysis identified simple rules with high accuracy to enable good out of sample generalization. In particular, it was possible to identify whether patients exhibited a typical cognitive profile of PD and whether they were compromised (or severely compromised) relative to frontal cortex cognitive abilities. Despite the high accuracies obtained, the present study suffers from a number of limitations; the major challenge encountered was that dependent variable, the performance on the AF consists, amongst others, of the predictors themselves. Additionally, due to the retrospective nature of our study, we were unable to report some clinical variables (e.g., disease severity, motor phenotype), and we lacked a control group, which would have allowed us to compare the performance of PD patients to that of healthy subjects.

In conclusion, our study shows that ML analysis of the classic semantic and phonemic verbal fluency test could represent an alternative and effective way of detecting accurate rules that enable the detection of set-shifting deficits in patients with PD.

## Supporting information

S1 Data (XLSX)
